# Detection of co-eluted peptides using database search methods

**DOI:** 10.1186/1745-6150-3-27

**Published:** 2008-07-02

**Authors:** Gelio Alves, Aleksey Y Ogurtsov, Siwei Kwok, Wells W Wu, Guanghui Wang, Rong-Fong Shen, Yi-Kuo Yu

**Affiliations:** 1 National Center for Biotechnology Information, National Library of Medicine, NIH, Bethesda, MD, 20894, USA; 2 Department of Mathematics, University of Maryland, College Park, MD, 20742, USA; 3 Proteomics Core Facility, National Heart, Lung, and Blood Institute, NIH, Bethesda, MD, 20892, USA

## Abstract

**Background:**

Current experimental techniques, especially those applying liquid chromatography mass spectrometry, have made high-throughput proteomic studies possible. The increase in throughput however also raises concerns on the accuracy of identification or quantification. Most experimental procedures select in a given MS scan only a few relatively most intense parent ions, each to be fragmented (MS^2^) separately, and most other minor co-eluted peptides that have similar chromatographic retention times are ignored and their information lost.

**Results:**

We have computationally investigated the possibility of enhancing the information retrieval during a given LC/MS experiment by selecting the two or three most intense parent ions for simultaneous fragmentation. A set of spectra is created via superimposing a number of MS^2 ^spectra, each can be identified by all search methods tested with high confidence, to mimick the spectra of co-eluted peptides. The generated convoluted spectra were used to evaluate the capability of several database search methods – SEQUEST, Mascot, X!Tandem, OMSSA, and RAId_DbS – in identifying true peptides from superimposed spectra of co-eluted peptides. We show that using these simulated spectra, all the database search methods will gain eventually in the number of true peptides identified by using the compound spectra of co-eluted peptides.

**Open peer review:**

Reviewed by Vlad Petyuk (nominated by Arcady Mushegian), King Jordan and Shamil Sunyaev. For the full reviews, please go to the Reviewers' comments section.

## Introduction

Protein identification and sequencing started almost sixty years ago. Some of the earliest techniques for protein identification required the protein of interest to be purified, and then digested with endoproteases, followed by sequencing of the resulting peptides. The protein was finally assembled [1] by joining sequenced peptides that have overlapping amino acids, as in the "shotgun-sequencing" of DNA. Technological improvements in the area of chromatography [2] and mass spectrometry (MS) [3-6], in particular tandem mass spectrometry (MS^2^), have revolutionized peptide sequencing and protein identification. In 1953, Frederick Sanger successfully sequenced the first protein, the bovine polypeptide hormone insulin. It took scientists over ten years to sequence insulin hormone of 51 residues using more than 0.1 Kg of protein [7]. Nowadays, laboratories using multidimensional protein identification technology (MudPIT) can partially sequence thousands of proteins in about 6–24 hours using only about 10–500 micrograms of sample [8]. It has also been repeatedly shown possible to identify 1500–2000 peptides within a digested complex protein mixture [9-12] from experiments using MudPIT with enhanced chromatography techniques.

Although this type of experiment is regularly used to identify proteins and to search for possible disease biomarkers [13,14], it is important to note that several aspects need to be improved upon in order to achieve optimal performance and maximal protein coverage. The challenging issues that have been reported and investigated include mass accuracy, precursor ion charge assignment, peptide retention time overlap, non-specific cleavage sites, number of missed cleavage sites, co-eluted peptides, acquisition time for a complete MS^2 ^scan, MS^2 ^spectrum signal to noise ratio, post-translational modifications, genomic single-nucleotide polymorphisms, incompleteness of protein databases, experimental reproducibility within and across laboratories, lack of common statistical standards for database search methods and others [15-21].

**Figure 1:**
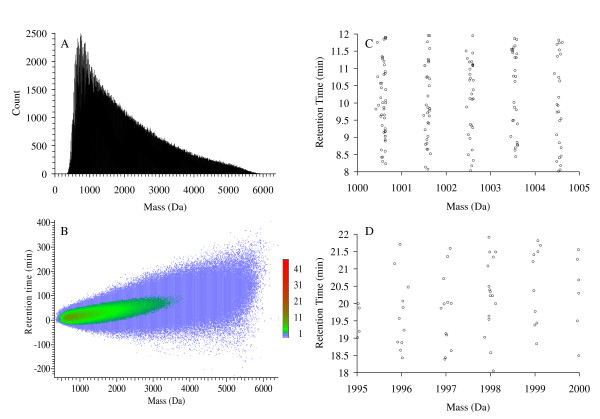
**Theoretical consideration of peptide co-elution**. (A) Frequency count (with bin width equals to five Dalton (Da)) of tryptically-digested peptides, allowing up to two miscleavages per singly-charged peptide, of all the 9393 Saccharomyces Cerevisiae proteins present in the NCBI's non-redundant protein database (04/23/2007). This results in a total of 771,753 unique peptides with molecular weights less than 6300 Da. (B) Theoretical retention times calculated for all of the 771,753 peptides [24]. The scale color code represents the number of peptides per grid but averaged over 25 grids (five mass bins × five retention time bins). Each grid has mass width of one Da and retention time width of 6 seconds. Since the retention time variation of a given peptide is typically much larger than 6 seconds, we may view the number encoded by color as the expected *minimal *number of co-eluted peptides whose mass differences are within one Da. This is due to the fact that a given peptide may appear in the mixture with charge states other than that is singly charged. Panels (C) and (D) result from zooming in on different regions of panel (B) to illustrate the minimal number of co-eluted peptides occurring within a small time window and with very similar masses. It is important to note that the complexity of the pictures above will significantly increase if one were to take into account post-translational modifications, incorrect cleavage sites, and multiple charge states.

In this study, we focus on the issue of co-eluted peptides. This problem often arises when one tries to analyze complex samples such as cell lysates, the focal point of shotgun proteomics aiming to quantify protein expressions in a cell subjected to various perturbations. To illustrate the intrinsic complexity of the problem, let us consider yeast which has a genome size of around 12 million nucleotides encoding approximately 6000 proteins. Upon taking all the yeast proteins present in the non-redundant database (nr) hosted by the National Center for Biotechnology Information (NCBI), digesting them *theoretically *using trypsin, and allowing up to two miscleavages, we obtain a large number of peptides whose molecular weight histogram is shown in Figure 1A. To mimic the effect of chromatographic separation on those peptides, we computed their retention times in reversed-phase HPLC [22,23] using the formula of Meek [24]. In Figure 1B, we show the spread and the density of retention times within each mass grid in a two dimensional heat map. Figure 1C and 1D are obtained from zooming in on two separate small regions in Figure 1B. The plots given in Figure 1 clearly show the complexity of the mixture and the frequent occurrences of co-eluted peptides in a given chromatography run. Although these plots are obtained theoretically, similar plots have been obtained experimentally [2,25].

Co-eluted peptides must have similar hydrophobicity and may be classified into four different types: type I co-eluted peptides are isobaric to each other and the mass spectrometer cannot distinguish them by using their molecular weights, type II co-eluted peptides are peptides that have close molecular weights which cannot be distinguished from one another due to limited machine resolutions, type III co-eluted peptides are peptides of different charge states that happen to have the same m/z and become indistinguishable from each other because of limited machine resolution, and type IV co-eluted peptides are peptides which have distinct molecular weights but have similar retention time. As suggested by the analysis in Figure 1, type I, II, and III co-eluted peptides must frequently occur when analyzing complex protein mixtures [17,18]. However, most analysis software are not pursuing identification of multiple true peptides. As for type IV co-eluted peptides, they are not commonly observed in MS^2 ^data due to the frequent use of data-dependent acquisition mode, which selects at each time only one of the most intense precursor ions of a previously acquired scan for further fragmentation. Selecting only the most intense precursor ion regardless of the presence of co-eluted peptides implies that fewer ions will be used for protein identification and quantification. Also, the low abundance proteins will be hard to identify and it becomes difficult to draw significant conclusions regarding protein expression levels.

To achieve better coverage of identified proteins and better sensitivity in detecting low abundance proteins, one may wish to consider selecting simultaneously multiple precursor ions for fragmentation. By allowing co-eluted peptides to be simultaneously fragmented, one increases the information collected per spectrum, albeit at the expense of introducing higher level of noise, and thus one may improve protein identification rate as well as the confidence in protein identification and quantification. In terms of collecting experimental data, the integrated MS multiplexed MS^2 ^data acquisition approach [26-29] seems only available for in-house machines but not for commercial MS analyzers in general. Other adaptations such as by changing the selecting window for the precursor ion [30] or perhaps by modifying the machine software might be other alternative route for pursuit.

The purpose of this study is to assess the readiness of software for analyzing this type of spectrum, hoping to encourage further hardware/software development to address this important issue. Although there exists software [31] specially designed to take into account co-eluting peptides, we do not include it in the present study. The scope of the current paper is to evaluate the likelihood for other generic database search engines in their present forms to gain in peptide identification rate from spectra of co-eluted peptides. Since the test spectra are superimposition of individual spectra that are commonly identified with high confidence by all search engines, in a way each search engine is tested under the best possible scenario. Generally speaking, a generic spectrum from co-eluted peptides may be noisier than those used in this study. To provide a better flow for the presentation, we have relegated information similar to what is presented in the main text to supporting information [Additional file 1]. For the remainder of the main text, we first describe in the materials section the starting data sets used, and we then describe the construction of the compound spectra needed for our study. The results and analyses are then presented, and we end with the concluding summary and outlook section.

## Materials

The database search tools included in this study are SEQUEST [32] (v27 rev12), Mascot [33] (v2.2), X!Tandem [34] (v2007.07.01.2), OMSSA [35](v2.0), RAId_DbS [36]. OMSSA, RAId_DbS and X!Tandem were installed for evaluation on the Biowulf cluster, a Linux parallel processing system with ≈ 3700 processors, of the National Institutes of Health (NIH). The runs using SEQUEST were done using the SEQUEST cluster of 18 processors at the Proteomics Core Facility of the National Heart, Lung, and Blood Institute (NHLBI), and the Mascot runs were done on the NIH's Helix system where eight processors are licensed to run Mascot software. Respective default search parameters were used for each of the database search methods except that for each method the allowed number of miscleavages was set to 3 and a minimum of 10 reported peptides per query was requested. A list of other parameters used is provided in Table 1.

**Table 1:** Database search tools parameters.

Search Method	PI m/z T	DI m/z T	NMCA
OMSSA	± 2.0 Da	± 0.8 Da	3
X!Tandem	± 2.0 Da	± 0.4 Da	3
SEQUEST	± 2.5 Da	± 1.0 Da	3
Mascot	± 2.0 Da	± 0.8 Da	3
RAId_DbS	± 3.0 Da	± 1.0 Da	Unlimited

In the first row of the table, "PI m/z T" stands for "parent ion m/z tolerance", "DI m/z T" stands for "daughter ion m/z tolerance" and "NMCA" stands for "number of miscleavages allowed."

For our study, we used a data set produced by the Proteomics Core Facility of the NHLBI of the NIH. This data set contains spectral data collected in profile mode only, and includes the following modes of instrument operation: LTQ/LTQ, FT/FT, TOF/TOF and LTQ/FT. This profile mode data set, previously described in reference [37], and henceforth referred to as the NHLBI data set, results from tryptic digestion of a mixture of eight proteins at three different concentrations and with the side chain of cysteine reduced with carbamidomethylation.

Bioworks (version 3.1) was used to extract peaks from the raw files of the data with no threshold imposed on the parent-ion ion count. For instrument modes such as LTQ/LTQ and LTQ/FT, one anticipates a low resolution in parent ion m/z and charge state determination. Therefore, we expand the spectrum charge state into both +2 and +3 when the charge state reported in the spectrum file was not +1. For the high resolution instruments such as FT/FT and TOF/TOF the charge state and parent-ion ion molecular weight obtained from the spectrum file were taken directly without any further expansion of charge state. The total number of spectra produced after extraction from the NHLBI set was 26159 and a detailed breakdown of the numbers of spectra by instrument type is given in Table 2. In order to have a balanced sampling among different instruments for the NHLBI data set, we created a new set of 10000 MS^2 ^spectra sampled as evenly as possible from spectra produced by different instruments. The breakdown of the numbers of spectra from different instruments is summarized in Table 3.

**Table 2:** Total number of MS^2 ^spectra obtained from NHLBI.

Molar	LTQ/LTQ	LTQ/FT	TOF/TOF	FT/FT
1000 nM	9190	840	1654	207
100 nM	9207	966	351	240
10 nM	2602	522	211	169

**Table 3:** Total number of MS^2 ^spectra used from NHLBI.

Molar	LTQ/LTQ	LTQ/FT	TOF/TOF	FT/FT
1000 nM	1624	840	1623	207
100 nM	1624	966	351	240
10 nM	1623	522	211	169

## Compound spectrum construction and use

Lacking a spectral data set that was produced by allowing more than one precursor ion per scan for further fragmentation, we computationally constructed compound spectra to mimic such a scenario. To produce a compound spectral data set with minimal bias towards any of the database search methods used, we proceeded as follows. The 10000 MS^2 ^spectra from the NHLBI set was first analyzed using all five database search methods mentioned earlier. The parameters in Table 1 were used for each method while searching, for each spectrum, in the nr protein database (480 Million residues) of the NCBI. For every spectrum, the reported results from each database search method were compared against a list of the theoretical peptides generated from the known proteins to determine whether a true peptide hit exists in those results. A query spectrum is classified as a member of the co-identifiable set provided that each search method is able to report, within a given *E*-value cutoff, at least one true peptide hit. To be more specific, the first criterion for a spectrum to be a member of the co-identifiable set is that all the five database search methods considered had to have a true positive identification for that spectrum, although not necessarily identifying the same peptide. The second criterion is that for each database search method the true positive peptide identified should have an *E*-value below 1. Because different database search methods report different confidence measures associated with the reported peptides, we have transformed the measure of each database search method to the proper *E*-value using a method reported earlier [37]. Table 4 summarizes the number of spectra contained in the co-identifiable set for the five database search methods considered. An Excel file [additional file 2] listing the spectra in the co-identifiable set is also provided. Each spectrum there is listed with the true peptide identified and the standardized *E*-values by turning the quality scores reported by the five search methods into *calibrated E*-values [37].

**Table 4:** Spectra co-identified by all database search tools considered with *E*-value less than 1.

Data	LTQ/LTQ	LTQ/FT	TOF/TOF	FT/FT
NHLBI	487/73	128/48	61/54	146/54

The first number represents the total number of peptides identified while the second number is the number of unique peptides identified.

We chose to include in the co-identifiable set only the NHLBI data set in order to minimize the occurrence of peptide co-elution so that each spectrum in the co-identifiable set will most likely contain only one true peptide. Because the NHLBI data set is generated by tryptically digesting only eight proteins, the likelihood of peptide co-elution has been greatly reduced compared to experiments using a complex protein mixture. A compound spectrum is constructed from combining spectra in the co-identifiable set. For each spectrum, we discretize the fragment ion masses by multiplying each mass by 1, 000 and then taking the integer part. Two different ways to combine spectra are investigated: sum (method 1) and max (method 2). In the former case, the intensity associated with a given mass index of the compound spectrum is given by summing over the chosen spectra all fragment ions with the same mass index. In the second method, for a given mass index in the compound spectrum the associated intensity is given by the maximum intensity among all fragment ions, within chosen spectra, with the same mass index. While combining spectra, we only allow mixing of spectra generated by the same instrument type.

Within each realization of compound spectrum construction, every spectrum in the co-identifiable set may be selected at most once for mixing. That is, within each realization we sample without replacement. As a concrete example, the procedure for combining spectra of the LTQ/LTQ instrument type is elaborated below. In the co-identifiable set, there are 487 spectra covering 73 unique peptides. To sample three spectra without replacement, one may obtain at most 487/3 ≈ 162 compound spectra per realization. To have a fair comparison of information retrieval when having different degrees of spectral mixing, the number 162 is used as the target number of spectra per realization for the LTQ/LTQ instrument type even when a compound spectrum contains only a single spectrum or two spectra. That is, we are simulating 162 MS^2 ^spectra when only the most intense, the two most intense, or the three most intense m/z precursor ions are selected for the second MS. Since it is impractical to evaluate all possible pairwise and triplet combinations of spectra, we only sample ten realizations of spectrum mixing. We also considered the case of mixing *only *spectra from distinct peptides and called this case unique peptide mixing. In this case, the selection of spectra deserves further explanation. For a given search method, a unique peptide *π *may be reported by search results from many different spectra. The representative spectrum of *π *for the given search method is chosen to be the one that reports *π *with lowest *normalized E*-value [37]. Basically, for each unique peptide *π*, a search method *M *is selecting for *π *the representative spectrum that is *easiest *for *M *to identify *π*. With this rule, different methods may end up selecting different sets of 73 spectra. Once the 73 spectra are selected for a given search method, one may then proceed to sample ten realizations of 24 ≈ 73/3 compound spectra, each made of either a single spectrum, two spectra, or three spectra as described earlier. Following the same logic, spectra mixing for other instruments are also done similarly and the corresponding numbers of spectra within each realization of sampling are summarized in Table 5.

**Table 5:** The number of spectra within each realization of sampling

Data	LTQ/LTQ	LTQ/FT	FT/FT	TOF/TOF
MAX1 (U)	162(24)	43(16)	49(18)	20(18)
MAX2/SUM2 (U)	324(48)	86(32)	98(36)	40(36)
MAX3/SUM3 (U)	486(73)	129(48)	147(54)	60(54)

The first number in the table is the number of compound spectra per realization of sampling when mixing spectra in the co-identifiable set. The number in parentheses is the number of compound spectra per realization of sampling when mixing only spectra of unique peptides (see text for more detail). The symbol "U" inside the parentheses stands for "unique peptide."

Once the compound spectra are assembled, we use each as a query spectrum to various search methods. For a doublet compound spectrum, the two precursor ion m/z's associated with the two spectra in the co-identifiable set will both be used for the search. That is, each program will process the same compound spectrum twice, once for each different m/z value for the precursor ion. The two reported hit lists are then merged together and sorted by *E*-value. Similarly, for a triplet compound spectrum, each program will process it three times, each time using a different parent ion precursor m/z. The three associated hit lists returned by a given search method are merged and again sorted by each entry's *E*-value. Apparently, the size of the final hit list for a compound spectrum is generally larger than that of a single spectrum in the co-identifiable set. For the proposed method to be useful, one first needs to make sure that the hit list expansion does not introduce an excessive number of false positives. A simple way to assess this aspect is to use Receiver Operating Characteristics (ROC) curves to describe the performance of a search method when using compound spectra. We will turn to this type of analysis in the next section.

## Analyses and results

Before analyzing the results, we briefly investigate the possibility of different peptides sharing multiple theoretical peaks in our test data. Counting contributions from all instrument types, the total number of unique peptides in the co-identifiable set is 110. For each of those 110 peptides, we calculate its corresponding theoretical b-type and y-type fragment masses with +1 charge. This results in 110 arrays of masses. We then exhaustively enumerate -between any two peptides and among any three peptides- the number of overlapped theoretical fragments. For two peptides, there are in total 110 × 109/2 = 5995 pairwise comparisons. For three peptides, there are in total 110 × 109 × 108/6 = 215820 comparisons. Any two (three) theoretical fragments from two (three) different peptides with mass difference(s) less than or equal to one Dalton (Da) contribute one count towards the number of shared peaks. The histograms of shared peaks are normalized by the total number of respective events to represent the probability of occurrence. Only the b-type and y-type fragments are used to compute the probability of fragment mass overlap because they usually are the most frequently observed in a spectrum with dominant intensities and are also the major series used for peptide identification by most database search methods.

**Figure 2:**
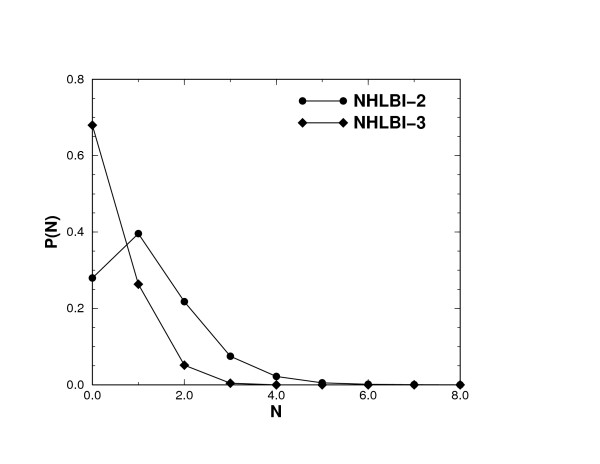
**Histograms of mass fragments overlap**. *P*(*N*) is the normalized histogram of the number of overlapping fragments of the b and y types between 2 or among 3 peptides from the 110 unique peptides in the co-identifiable set. The peak at *N *= 1 for two peptides is due to that fact that the C-terminal amino acid is either K or R, resulting in a large probability for two peptides to have the same y-1 peaks and thus contribute significantly to the histogram at *N *= 1. This artifact diminishes as we look into the shared peaks among three peptides since the chance for peptides to share the same C-terminal amino acid decreases with the number of peptides.

**Figure 3:**
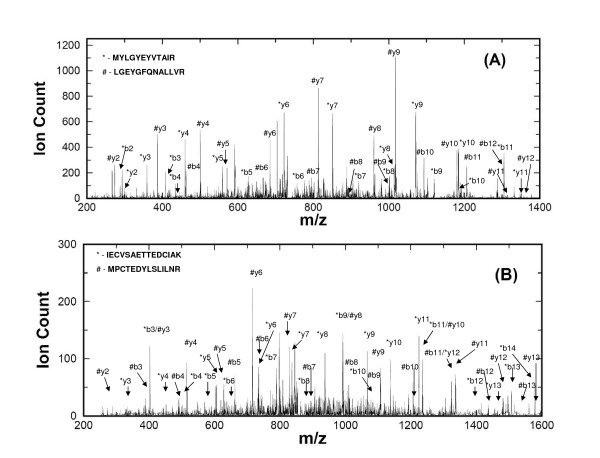
**Example spectra of peptide co-elution**. (A) and (B) are experimental spectra of two co-eluted peptides simultaneously identified by RAId_DbS, OMSSA, SEQUEST and Mascot.

As shown in Figure 2, the probability for two peptides to share three or more peaks of the b-type and y-type is less than 5% and the probability becomes less than 1% for three randomly selected peptides to share three or more theoretical peaks. These probabilities indicate that a generated compound spectrum in general does not contain many peaks that may be used to identify several true peptides. Consequently, the identification of multiple peptides is not due to shared fragments between peptides. Two experimental spectra containing type II co-eluted peptides are shown in Figure 3. There are indeed very few b and y fragment overlaps in either spectrum, consistent with the statistics collected from our compound spectra. This experimental data, in some way, justify our compound spectra construction strategies. In both experimental spectra shown, the existence of more than one true peptide hit is confirmed by more than one search method.

As a result, the compound spectra generated are very random in terms of peaks used to identify the true peptides and all other peaks present in a given compound spectrum may be regarded as noise peaks. Hence, the data set generated is suitable for testing the ability of different database search methods to identify true positive peptides when given a complex spectrum where the signal to noise ratio has been decreased due to the introduction of several co-eluted precursor ions.

### *E*-value distributions for the co-identifiable spectra

**Figure 4:**
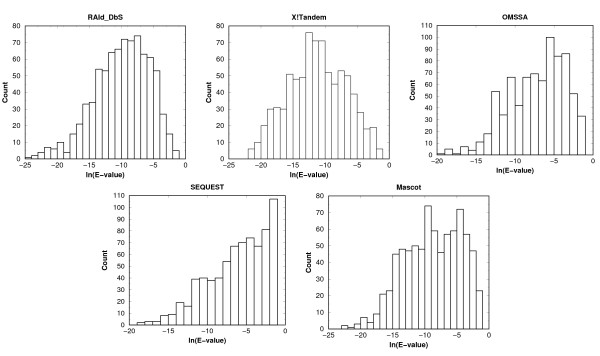
***E*-value distributions of true peptides identified**. The *E*-value distributions of the true peptides identified from spectra in the co-identifiable set. The average *E*-values are: RAId_DbS (*μ *= 0.05), X!Tandem (*μ *= 0.025), Mascot (*μ *= 0.04), OMSSA (*μ *= 0.03) and SEQUEST (*μ *= 0.05).

To ensure that the co-identifiable set used for generating the compound spectra does not bias towards any given database search method, we plotted the *E*-value distribution for those co-identifiable peptides. The *E*-value distributions for the database search methods tested all have average *E*-values less than 0.05 and all are distributed over a wide range (see Figure 4), indicating minimal bias towards any given method tested.

### Performance assessment and analyses

**Figure 5:**
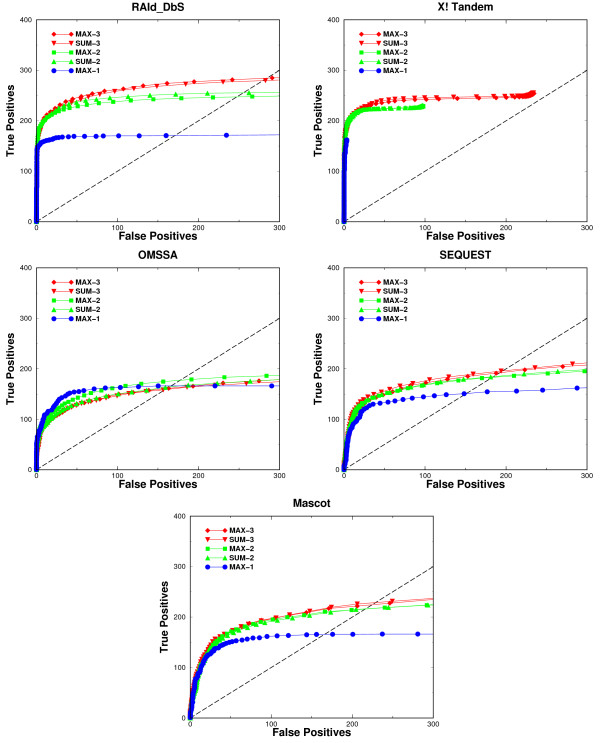
**ROC analyses of compound spectra**. ROC curves constructed from analyzing the compound spectra of LTQ/LTQ type. In the figure legend, MAX-1 represents the ROC curve from analyzing single peptide spectra sampled directly from the co-identifiable set, MAX-2 (SUM-2) and MAX-3 (SUM-3) are the ROC curves from analyzing compound spectra formed by combining respectively two and three single-peptide spectra in the co-identifiable set. The symbol "MAX" in the legend indicates that each compound spectrum is obtained by taking at every mass grid the maximum intensity among the peaks of the spectra combined, while "SUM" in the legend indicates the compound spectra are obtained through summing at every mass grid the intensities of peaks of the spectra to be combined. The Arabic numbers 2 and 3 in the legend indicate the number of spectra combined to form a compound spectrum.

**Figure 6:**
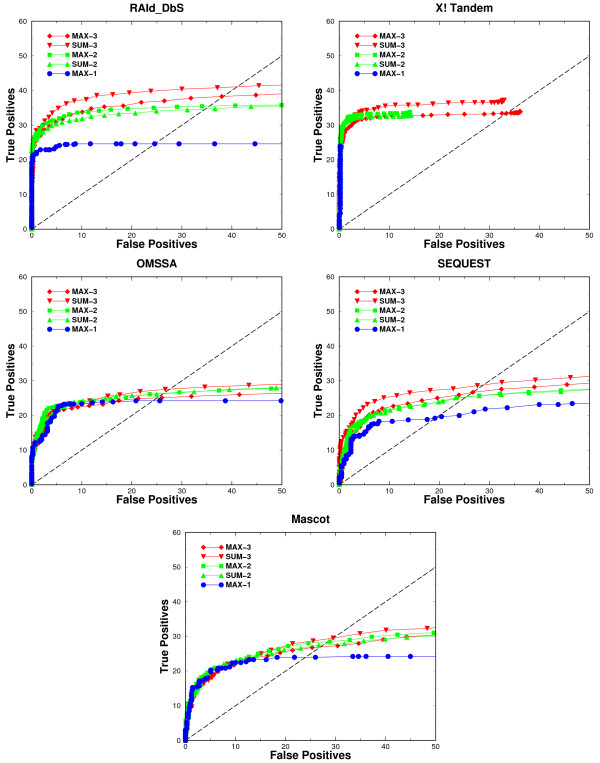
**ROC analyses of compound spectra of distinct peptides**. ROC curves constructed from analyzing the compound spectra formed by combining a number of unique peptide spectra of the LTQ/LTQ type. In the figure legend, MAX-1 represents the ROC curve from analyzing single peptide spectra sampled directly from the co-identifiable set, MAX-2 (SUM-2) and MAX-3 (SUM-3) are the ROC curves from analyzing compound spectra formed by combining respectively two and three single-peptide spectra in the co-identifiable set. The symbol "MAX" in the legend indicates that each compound spectrum is obtained by taking at every mass grid the maximum intensity among the peaks of the spectra combined, while "SUM" in the legend indicates the compound spectra are obtained through summing at every mass grid the intensities of peaks of the spectra to be combined. The Arabic numbers 2 and 3 in the legend indicate the number of spectra combined to form a compound spectrum.

We have used Receiver Operating Characteristics (ROC) curves (Figures 5 and 6) to assess the performance of each of the five search engines using the compound spectra derived from the LTQ/LTQ data in the co-identifiable set. Each ROC curve plotted is the average result over ten different realizations of sampling. The corresponding ROC curves (for OMSSA, X!Tandem, and RAId_DbS) from analyzing compound spectra generated by TOF/TOF, LTQ/FT and FT/FT instruments are shown in the supplementary material. For a given search method, an individual panel containing five ROC curves is used to summarize the results. The five ROC curves in each panel correspond to the performance of the search method when the compound spectra contain one, two, or three co-identifiable peptides. Since the compound spectra mimicking either two or three co-eluted peptides are constructed in two ways, we ended up having five ROC curves in total. To show how much one may gain in terms of true positive retrieval, we show the ROC curve from analyzing simulated co-eluted spectra and compare it against the baseline ROC curve, obtained from directly analyzing co-identifiable spectra without mixing. Because each spectrum in the co-identifiable set contains one true positive peptide identified by all search methods, all the baseline ROC curves should reach a common maximum.

For each search method tested, using either the sum of intensities (method 1) or the max intensity (method 2) to generate the compound spectrum do not result in any appreciable variance in the ROC curves. This indicates that most current database search methods are robust in retaining the information used to identify peptides. In other words, the data pre-processing used by most database search methods such as spectrum normalization, peak selection and data centroidization do not lose the information needed for the identification of true peptides.

For X!Tandem, RAId_DbS, SEQUEST and Mascot, as shown in the ROC curves of Figure 5, there is a consistent identification improvement when analyzing spectra mimicking more co-eluted peptides (up to three). OMSSA's performance using spectra mimicking multiple co-eluted peptides is comparable to its baseline, except at low number of false positives region where the ROC curve for two co-eluted peptides is worse than the baseline ROC curve and the ROC curve of three co-eluted peptides is worse than that of two co-eluted peptides. OMSSA's performance trend may be related to its use of a Poisson probabilistic background model where a random peak being counted as one of the *b *or *y *peaks is assumed to be a Poisson event. Since we are combining spectra acquired in profile mode, which intrinsically report a lot more peaks than data collected in centroid mode, any compound spectrum may be very rich and complex with many strong peaks. This might not agree with the probabilistic model assumed by OMSSA and somehow compromise its performance. We believe that OMSSA should also be able to gain improvement in ROC analysis provided that one uses centroid data, although we currently don't have analysis to support this conjecture.

The details of Mascot's probabilistic model are not publicly available, making it hard to comment on what might happen when a different data acquisition mode is used. We only know that Mascot's probabilistic model is similar to the model used in MOWSE [38]. In the case of SEQUEST one would not expect significant dependence on the data type used as it uses correlation as a measure for peptide identification between theoretical fragments and experimental fragments and in principle it will be able to identify true peptides as long as the information necessary for identification is present in the spectrum and the spectrum is not over populated with strong peaks, which is the case for the spectra used. X!Tandem and RAId_DbS both fit the score distribution generated from scoring experimental spectrum against peptides in a given database. While X!Tandem uses an exponential [39] to approximate the tail of the score distribution, RAId_DbS's score distribution has a theoretical foundation and accommodates skewness and finite sample effect [36]. Neither method is expected to have substantial change in performance when using a different data type.

**Figure 7:**
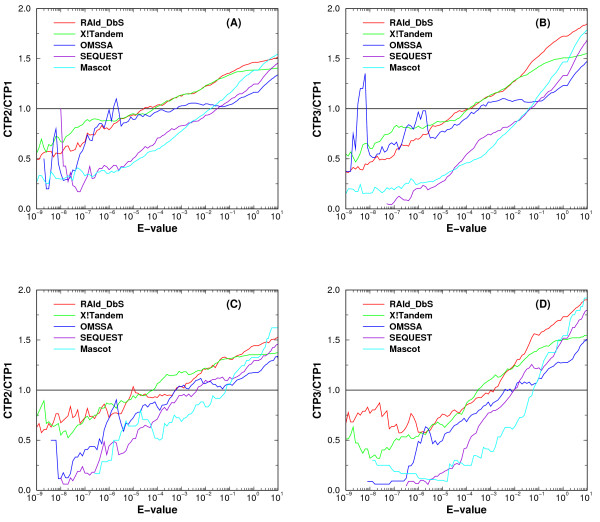
**Cumulative identification ratio versus *E*-value cutoff for compound spectra**. Panel A/B and C/D displays the cumulative identification ratio as a function of *E*-value cutoff for the five database search tools when analyzing compound spectra constructed by the SUM2/SUM3 method while combining the LTQ/LTQ single-peptide spectra (A/B) and unique-peptide spectra (C/D) in the co-identifiable set. The symbols CTP1, CTP2 and CTP3 correspond respectively to the cumulative number of true positives (TP) identified with *E*-value equal to or smaller than the specified cutoff when analyzing single-peptide spectra, compound spectra of two peptides, and compound spectra of three peptides.

Upon inspection of the ROC curves for the five database search methods tested, it seems that the ROC curves for the compound spectra each resulting from 2 or 3 co-eluted peptides always climb above the ROC curve for spectra each resulting from only a single peptide. This implies that there would be no loss in protein coverage by sending simultaneously the 2 or 3 most intense precursor ions to the second MS to generate a convoluted spectrum. Since the transformed *E*-value [37] now may serve as a common statistical standard across different search methods, one may wish to understand how the statistical significance assignment of the identified peptides is impacted by using a convoluted spectrum. To this end, we first define the *cumulative identification ratio *as the ratio of the cumulative number of true peptides identified from compound spectra (each resulting from 2 or 3 co-eluted peptides) to that from the co-identifiable spectra (each resulting a single peptide). We then plotted the cumulative identification ratio against the *E*-value cutoff (Figure 7). As shown in Figure 7, all search method eventually go above the horizontal line *y *= 1, indicating an increase in peptide coverage. One also observes that the ratio at low *E*-value is smaller than one. Such a result is expected since each compound spectrum becomes quite complex due to spectrum mixing and the higher noise level makes it harder for a true positive peptide's score to be significantly higher than the background. As a consequence, a true peptide hit here may be assigned a higher *E*-value.

## Discussion

It is worth pointing out that RAId_DbS and X!Tandem both climb above the line *y *= 1 at a lower *E*-value and they also reach a greater number of true positive identified when compared to the other three methods. As an example, for *E*-value ≤ 0.01, RAId_DbS and X!Tandem attain an increase of around 30% in the number of true positives identified and an increase up to 50% or better in the number of true positives identified when *E*-value ≤ 0.1 is used as the criterion. Table 6 provides the percentage increase in the number of true peptides identified over the baseline *y *= 1.

**Table 6:** The percentage of the total number of peptides identified, allowing multiple co-eluted peptides per spectrum, when compared to that of the single peptide identification at a fixed *E*-value.

	CTP2/CTP1		CTP3/CTP1	
	E = 10^-2^	E = 10^-1^	E = 10^-2^	E = 10^-1^

RAId_DbS (U)	1.22(1.21)	1.40(1.31)	1.26(1.26)	1.53(1.55)
X!Tandem (U)	1.24(1.22)	1.34(1.31)	1.27(1.25)	1.44(1.41)
Mascot (U)	0.95(0.83)	1.15(1.05)	0.87 (0.64)	1.13(1.13)
SEQUEST (U)	0.95(1.10)	1.10(1.12)	0.92(1.01)	1.11(1.22)
OMSSA (U)	1.05(1.06)	1.02(1.06)	1.11(1.01)	1.08(1.12)

To the right of each search method, the first number in each grid is the ratio of number of identified peptides using the compound spectra to that using single peptide spectra at various *E*-value cutoff. The number in parentheses is the ratio obtained by using only compound spectra constructed from mixing the spectra of unique peptides. Here the symbol "U" stands for "unique peptide". The symbols CTP1, CTP2 and CTP3 correspond respectively to the cumulative number of true positives (TP) identified with *E*-value equal to or smaller than the specified cutoff when analyzing single-peptide spectra, compound spectra of two peptides, and compound spectra of three peptides.

In the ideal scenario, if one were to analyze 1, 000 experimental spectra using RAId_DbS or X!Tandem, by selecting the two most intense ions for second MS instead of just the most intense ion, with *E*-value ≤ 0.01 one anticipates increasing number of identified peptide by 30% with the number false positives increasing by approximately 10. And this identification increase may rise to 50% if one is willing to use *E*-value ≤ 0.1 as a cutoff resulting in approximately 100 more false positives in this case. The increase of 100 false positives may seem more than one is willing to accept. However, in the ideal situation where each of the 1, 000 spectra was identified with a true positive, there would be 500 more true peptides identified and the increase of 100 false positives may become acceptable. Therefore, where to set the *E*-value cutoff to gain more true positives without introducing an unacceptable number of false positives crucially depends on the identification rate for single-peptide spectra. Consequently, protein identification and quantification might benefit from analyzing spectra of co-eluted peptides when proper *E*-value cutoff is used. However, it is also true that not all database search methods have a significant increase in the number of true positives identified at low *E*-values. Nevertheless, the number always exceeds the baseline when a larger *E*-value cutoff is considered. Therefore the problem in analyzing convoluted spectra is not that the true positives can't be found, but rather, their assigned *E*-values might be higher than one wishes due to the high noise level introduced by spectral mixing.

A possible way to reduce the effect of noise is to construct a revised spectrum by subtracting from the convoluted spectrum of two or three co-eluted peptides the theoretical fragments of the lowest *E*-value peptide reported prior to further processing. As an example, for a convoluted spectrum made of two co-eluted peptides, one has two precursor m/z's to search in the database. The peptide hit with lowest *E*-value will correspond to an m/z, which we call the first m/z. One may then generate this peptide's theoretical fragments and subtract them from the convoluted spectrum to form a revised spectrum. The revised spectrum is then used as query to search for candidate peptides corresponding to the second m/z. It was not the goal of this study to provide a method to improve the reported *E*-value of co-eluted peptides. Instead, we evaluate the readiness of software to handle such spectra, hoping to encourage developers of database search methods to enhance their software capabilities in handling co-eluted spectra.

## Concluding summary and outlook

In our study we computationally investigated the possibility of using current database search methods to identify peptides from the superimposed MS^2 ^spectra of 2 and 3 peptides. Based on our theoretical analysis (see Figure 1), peptides will be co-eluted all the time during a large scale HPLC/MS proteomics experiment. Even if one is able to identify from each spectrum a correct peptide, information retrieval needed for protein coverage and protein expression level assessment may still be improved by taking into account the possibility of peptide co-elutions.

To mimic the spectra resulting from co-eluted peptides, we mixed single peptide spectra. In our case, because we use a data set generated by only eight proteins, the likelihood of peptide co-elution has been greatly reduced, justifying our assumption that the co-identifiable set mainly contains single peptide spectra. We have employed two different methods of spectrum mixing and their results are very similar. The main finding in this paper is that it is possible to increase the information retrieval in peptide identification if one were to consider analyzing type IV co-eluted peptides even with current database search methods. Clearly, there is still room for improvement for every software package in terms of identifying type IV co-eluted peptides.

However, it is worth remarking that type I to type III co-eluted peptides are already abundant in the current experimental set up. An increase in protein coverage may already be attainable if software developers can look into this aspect and enhance their softwares' detection capabilities in this regard. Even before considering type IV co-eluted peptides, it may already benefit the community if the shackles of "one true peptide per spectrum" are removed. Of course, any statistical assessment method developed should also take into account the possibility of having more than one correct peptide per spectrum. It is our hope that this study will stir the interest of software developers to include in their search methods features that allow for better detection of co-eluted peptides.

## Competing interests

The authors declare that they have no competing interests.

## Authors' contributions

GA, AO and YKY designed the research. GA, AO and SK carried out the research. GA, AO, SK and YKY analyzed the results. WW, GW and RFS did the experiment and participated in data analysis. GA and YKY wrote the paper. All the authors have read and approved the final version of the manuscript.

## Reviewers' comments

### Reviewer's report 1

Review by Vlad Petyuk, nominated by Arcady Mushegian, Biological Separations and Mass Spectrometry Biological Science Division, Pacific Northwest National Laboratory.

1) The authors address the problem of concurrent fragmentation of co-eluting peptides. I agree that this is a problem; however its extent is not clear. The justification provided in the manuscript, in particular on Figure 1, is based on in *silico *tryptic digestion of yeast proteome. That assumes that all the proteins are expressed in a given condition and present in about equal amounts. Both of the assumptions are likely to be false for real samples. First, even for a simple organism such as E. coli, there is only a subset of proteome which is expressed at a given moment. Second, if peptides significantly differ in abundance the low abundance peptide(s) will be masked by noise in both MS and MS/MS spectra and high abundant peptide will appear as single peptide per spectrum the database search software. Thus, I would encourage estimating the percentage of MS/MS spectra resulting from simultaneous peptide fragmentation, and indeed containing fragment ions of both peptides, on some real LC-MS/MS dataset of complex sample analysis (for example MudPIT experiment). Actual numbers should help to understand the significance of the problem. However, certain not yet widely used approaches (referenced as #27 and #30) utilize broadband fragmentation instead of more common +/- 1.5 m/z tolerance window around the parent ion. In such cases consideration of concurrent fragmentation can not be ignored.

2) The other question would be regarding the construction of compound spectra. Has the parent ion mass to charge ratio been accounted for realization of compound spectra? If the MS/MS spectra were combined regardless the parent ion information, then it does not reflect the typical real situation of LC-MS/MS experiments, but it rather imitate broadband fragmentation situation. The compound spectra were searched 2 or 3 times, each time assuming different parent ion mass. Again, in reality MS/MS search engines search the spectra only once, as it is only one parent mass associated with a spectrum. For the case of broadband (aka data-independent) or wide parent ion mass tolerance fragmentation there are also no set of defined distinct set of parent ion masses associate with a spectrum. What was studied is the scenario of not yet widely used data-dependent multiplexed fragmentation (ref #28) classified as type IV in the presented manuscript.

3) Authors, note that they use MS/MS spectra collected in only profile mode for this study. However centroid mode is common mode for collecting MS/MS data.

4) I strongly encourage using conventional instrument names and modes of their operation. This is significant source of confusion. There are no such instruments as LTQ/LTQ and FT/FT. I believe what is described is acquisition of MS and MS/MS spectra in the low resolution mode in the ion trap and in high resolution mode in ICR cell. For example authors describe instrument set up LTQ/FT as low resolution in parent m/z and charge state determination. I believe this is an error resulting from mixing unconventional names of instrument operating modes with actual instrument names. It would be quite odd to run LTQ FT Ultra hybrid mass spectrometer instruments in low resolution ion trap mode for MS spectra acquisition.

5) It seems to me that the call for development of software capable of handling convoluted MS/MS spectra has already been at least partially addressed. Please provide any comments on the manuscript entitled "ProbIDtree: An automated software program capable of identifying multiple peptides from a single collision-induced dissociation spectrum collected by a tandem mass spectrometer" published by Aebersold group in Proteomics 2005.

6) If possible I would encourage combining figures with highly redundant legends for sake of comprehension. For example legends of Figure 7 and Figure 8 differ only in one word.

Overall it is a valuable study aimed at evaluation of commonly used MS/MS search engines. It is certainly good news that most of the search engines do not fail in the cases of convoluted spectra and moreover may identify multiple peptides if multiple parent ion masses provided. However it is achieved, as author notes, at the cost of statistical confidence. I believe exploring possibilities of both data-dependent and data-independent multiplexed fragmentation approaches and tuning the scoring schemes and statistics to deal with such spectra has a high chance of generating a good return on investment.

### Author's response

1) The example provided in Figure 1 is used to demonstrate the complexity of a cell lysate sample. Figure 1 actually is a simplified picture of a cell lysate sample as it does not account for: post translation modifications, single nucleotide polymorphism and it only includes correct trypic digested sites allowing up to two miscleavage sites. We have included two references [2,26] that contain similar plots for real biological sample similar to the one shown in Figure 1.

Regarding the second reviewer comment, indeed for a *synchronized cell sample*, only a subset of the proteome is expressed at a given moment. In reality, most of the time the cell sample used are not synchronized, and one is looking at the time averaged distribution of the expressed proteins. We agree with the reviewer regarding low abundant peptides. If peptides are present in the sample at concentration levels that makes them indistinguishable from noise those peptides probably will not be identified, and if identifiable they will in general have a low confidence score associated with their identification.

We are currently considering to conduct a global study of co-eluted peptides of type I, II, III present in complex mixtures as suggested by the reviewer. We actually have the result from a small data set composed of a mixture of 7 proteins containing 6,734 spectra analyzed using RAId_DbS. In this data set we observed a total of 93 spectra having two true positives [40], which was the motivation for the current study to evaluate how well different database search methods perform given a convoluted MS/MS spectrum.

2) Our protocol of compound spectral construction mimicks type IV (data-dependent multiplex) co-eluted peptides the most, but it also include types I, II, and III. The broad band approach, where spectrum were searched with a wide range of m/z, is one way to capture co-eluted peptides but is not what we intended to study here. The scope of this study is to assess in general how well different database search methods perform given a number of convoluted MS/MS spectra regardless of whether the convoluted spectra is of the type I, II, III or IV, but not of the broad band type. The compound spectra were searched multiple times, based on the (m/z)s of the parent ions mixed similar to the data-dependent multiplex method.

3) We did not used the data collect in centroid mode because these are processed spectra using some denoise (centroid) software package that comes with the instrument, with little reference, if any, to how the centroid spectra are produced. Also many peaks get removed during the centroid process which can affect peptide identifications especially when one is trying to identify multiple co-eluted peptides from a convoluted MS/MS spectrum.

4) We thank the reviewer for pointing out that LTQ/LTQ (or FT/FT or LTQ/FT or FT/LTQ) refers to the mode of instrument operation for spectra acquisition. The notation used XXX/YYY (LTQ/LTQ or FT/FT or LTQ/FT or FT/LTQ) refers to the mode of data acquisition for MS and MS/MS spectra. For example, FT/LTQ indicates survey MS spectra were acquired by the FT-ICR detector and MS/MS spectra were acquired in the LTQ linear ion trap. Also a Finnigan LTQ FT hybrid mass spectrometer (Thermo Scientific, San Jose, CA) was used to acquire spectra for the following modes of operation: LTQ/FT, LTQ/LTQ, and FT/FT. The TOF/TOF mode of spectra acquisition was performed with an Applied Biosystems 4700 Proteome Analyzer MALDI TOF/TOF (Foster City, CA).

The purpose of using the LTQ/FT mode was to evaluate the different database search methods retrieval using as many instrument combinations as possible.

5)We thank the referee for pointing out this reference. We have now included this reference in the introduction.

6) We have merged Figure 8 with Figure 7 as suggested.

### Reviewer's report 2

Review by King Jordan, School of Biology, Georgia Institute of Technology.

I support publication of this manuscript in Biology Direct.

### Reviewer's report 3

Review by Shamil Sunyaev, Harvard Medical School.

This interesting manuscript challenges current strategy of shotgun proteomics in selecting precursor ions. The authors demonstrate that all current computational methods for peptide identification (possibly, with the exception of OMSSA) are capable of identifying co-eluted peptides. This computational experiment suggests new ways of the proteomic analysis which would not be limited to a few highest intensity parent ions and will include multiple co-eluted peptides.

## Supplementary Material

Additional file 1 Supporting information for Detection of Co-eluted Peptides using Database Search Methods. ROC curves and cumulative ratio plots of X!Tandem, RAId_DbS and OMSSA from analyzing profile mode spectra generated by LTQ/FT, TOF/TOF and FT/FT instruments. Click here for file

Additional file 2 Profile co-identifiable-spectra. This file lists the spectra in the co-identifiable set. Each spectrum is listed with the true peptide identified and the standardized *E*-values by turning the quality scores reported by the five search methods into *calibrated E*-values [37]. Click here for file
