# Supplementary material for: Detection of co-eluted peptides using database search methods
Source: Biol Direct. 2008 Jul 2;3:27. doi: 10.1186/1745-6150-3-27 (PMC2483259; doi:10.1186/1745-6150-3-27)
Supplement: Additional file 1 — Supporting information for Detection of Co-eluted Peptides using Database Search Methods. ROC curves and cumulative ratio plots of X!Tandem, RAId_DbS and OMSSA from analyzing profile mode spectra generated by LTQ/FT, TOF/TOF and FT/FT instruments. [file 1745-6150-3-27-S1.pdf]

Supporting information for Detection of Co-eluted Peptides using Database Search Methods

Gelio Alves[a], Aleksey Y. Ogurtsov[a], Siwei Kwok[b], Wells W. Wu[c], Guanghui Wang[c], Rong-Fong Shen[c] and Yi-Kuo Yu[a*]

[a]National Center for Biotechnology Information, National Library of Medicine, National Institutes of Health, Bethesda, MD 20894

[b]Department of Mathematics, University of Maryland, College Park, MD, 20742.

[c]Proteomics Core Facility, National Heart, Lung, and Blood Institute, National Institutes of Health, Bethesda, MD 20892

[*]To whom correspondence should be addressed: yyu@ncbi.nlm.nih.gov

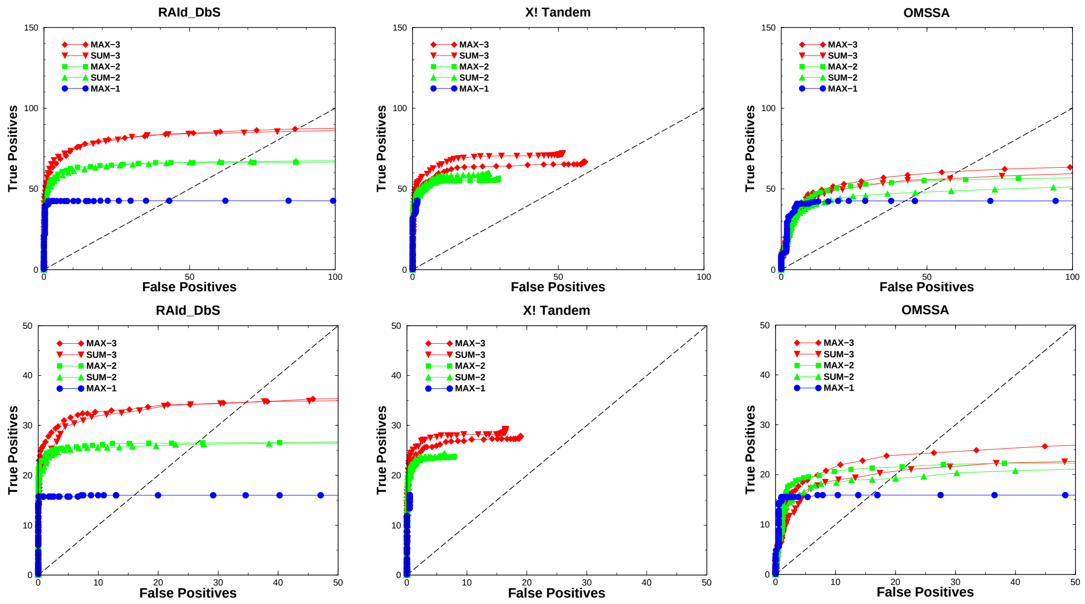

Figure 8: In the first row are ROC curves constructed by analyzing the compound spectra from combining single-peptide spectra of LTQ/FT type in the co-identifiable set. In the second row are the ROC curves constructed by analyzing the compound spectra from combining unique-peptide spectra of LTQ/FT type in the co-identifiable set. In the figure legend, MAX-1 represents the ROC curve from analyzing spectra of one peptide sampled directly from the co-identifiable set, MAX-2 (SUM-2) and MAX-3 (SUM-3) are the ROC curves from analyzing compound spectra by combining respectively two and three peptide spectra in the co-identifiable set. The symbol "MAX" in the legend indicates that each compound spectrum is obtained by taking at every mass grid the maximum intensity among the peaks of the spectra combined, while "SUM" in the legend indicates the compound spectra used are obtained by summing at every mass grid the intensities of peaks of the spectra combined. The arabic numbers 2 and 3 in the legend indicate the number of spectra combined to form a compound spectrum.

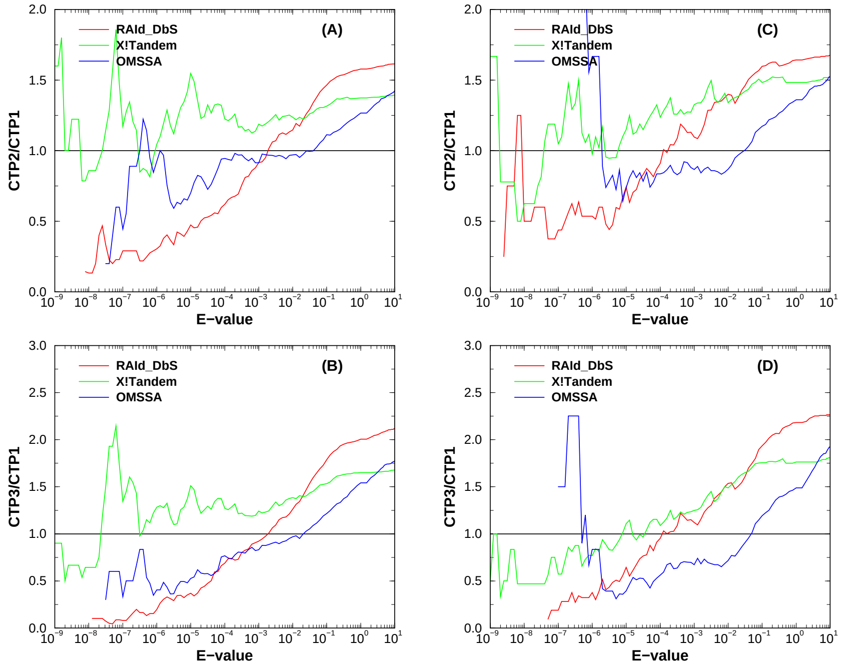

Figure 9: The cumulative identification ratio from analyzing compound spectra made by combining two (A) or three (B) LTQ/FT spectra in the co-identifiable set. The compound spectra are constructed using the SUM2 and SUM3 methods. Panels (C) and (D) show essentially the same content except that only unique-peptide spectra are combined to form compound spectra. The symbols CTP1, CTP2 and CTP3 correspond respectively to the cumulative number of true positives (TP) identified with E-value equal to or smaller than the specified cutoff when analyzing single-peptide spectra, compound spectra of two peptides, and compound spectra of three peptides.

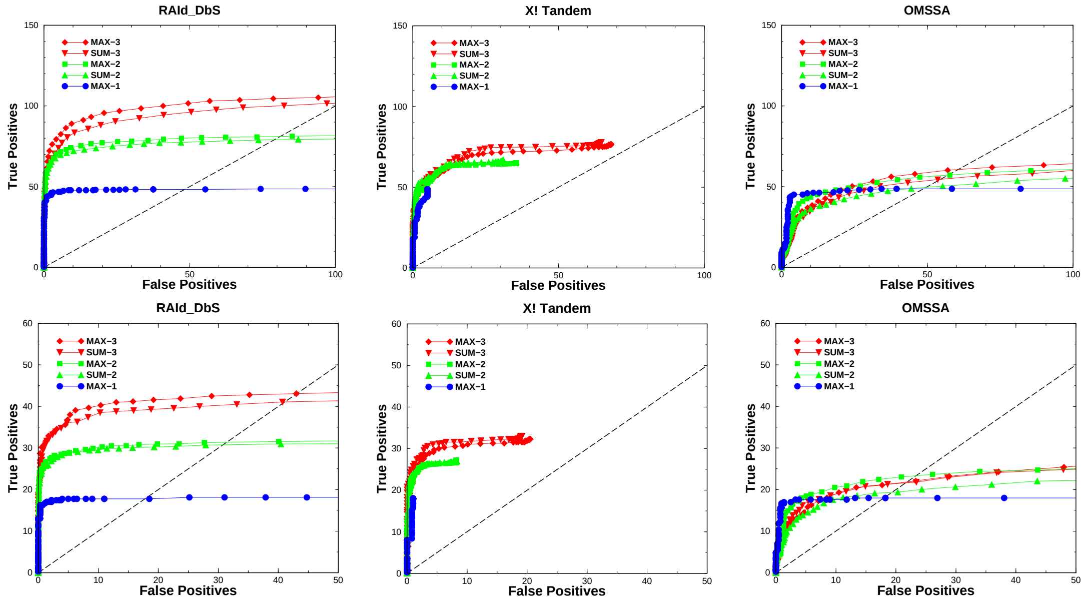

Figure 10: In the first row are ROC curves constructed by analyzing the compound spectra from combining single-peptide spectra of FT/FT type in the co-identifiable set. In the second row are the ROC curves constructed by analyzing the compound spectra from combining unique-peptide spectra of LTQ/FT type in the co-identifiable set. In the figure legend, MAX-1 represents the ROC curve from analyzing spectra of one peptide sampled directly from the co-identifiable set, MAX-2 (SUM-2) and MAX-3 (SUM-3) are the ROC curves from analyzing compound spectra by combining respectively two and three peptide spectra in the co-identifiable set. The symbol "MAX" in the legend indicates that each compound spectrum is obtained by taking at every mass grid the maximum intensity among the peaks of the spectra combined, while "SUM" in the legend indicates the compound spectra used are obtained by summing at every mass grid the intensities of peaks of the spectra combined. The arabic numbers 2 and 3 in the legend indicate the number of spectra combined to form a compound spectrum.

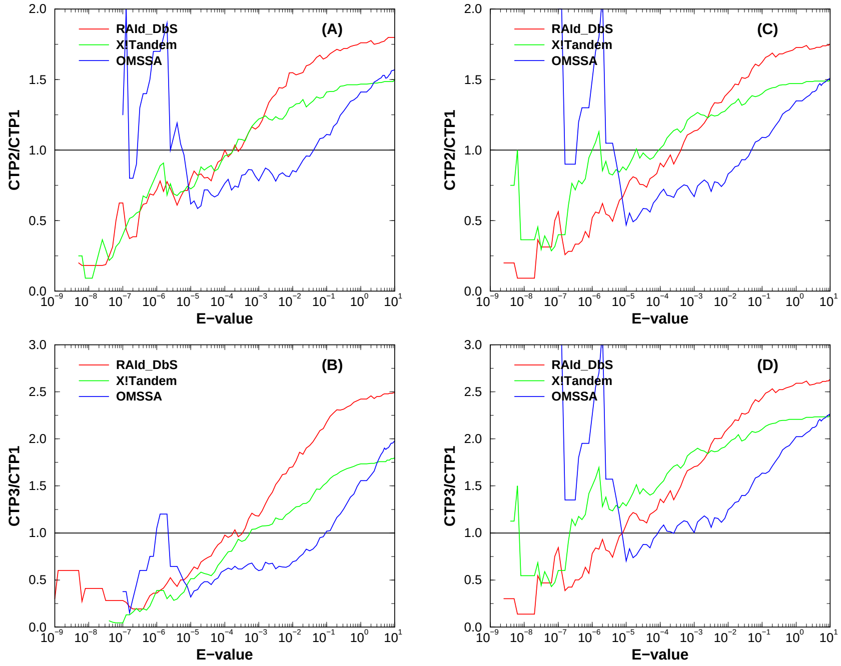

Figure 11: The cumulative identification ratio from analyzing compound spectra made by combining two (A) or three (B) FT/FT spectra in the co-identifiable set. The compound spectra are constructed using the SUM2 and SUM3 methods. Panels (C) and (D) show essentially the same content except that only unique-peptide spectra are combined to form compound spectra. The symbols CTP1, CTP2 and CTP3 correspond respectively to the cumulative number of true positives (TP) identified with E-value equal to or smaller than the specified cutoff when analyzing single-peptide spectra, compound spectra of two peptides, and compound spectra of three peptides.

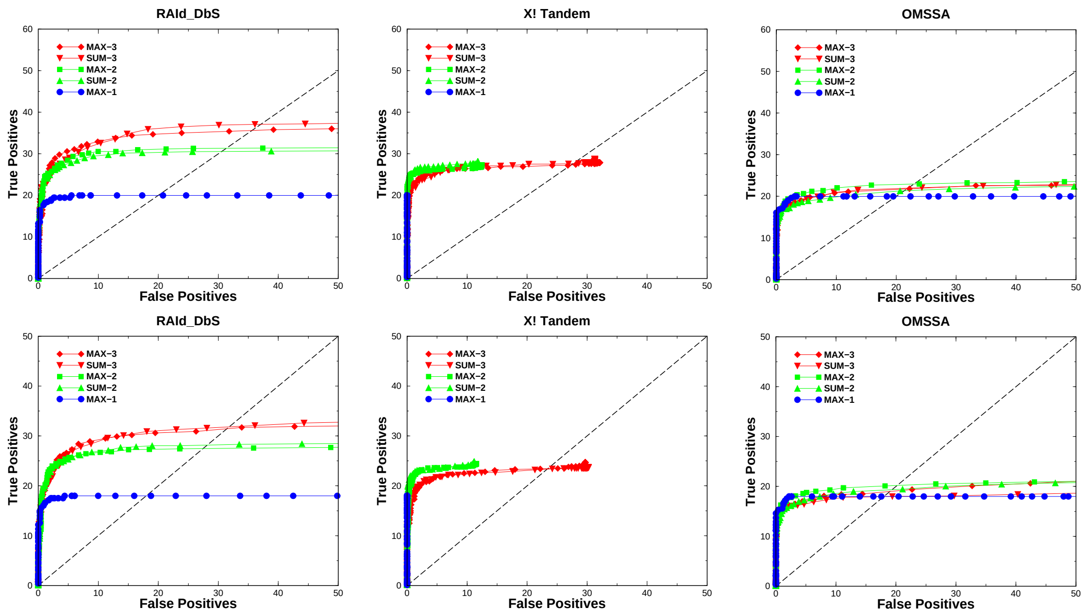

Figure 12: In the first row are ROC curves constructed by analyzing the compound spectra from combining single-peptide spectra of TOF/TOF type in the co-identifiable set. In the second row are the ROC curves constructed by analyzing the compound spectra from combining unique-peptide spectra of LTQ/FT type in the co-identifiable set. In the figure legend, MAX-1 represents the ROC curve from analyzing spectra of one peptide sampled directly from the co-identifiable set, MAX-2 (SUM-2) and MAX-3 (SUM-3) are the ROC curves from analyzing compound spectra by combining respectively two and three peptide spectra in the co-identifiable set. The symbol “MAX” in the legend indicates that each compound spectrum is obtained by taking at every mass grid the maximum intensity among the peaks of the spectra combined, while “SUM” in the legend indicates the compound spectra used are obtained by summing at every mass grid the intensities of peaks of the spectra combined. The arabic numbers 2 and 3 in the legend indicate the number of spectra combined to form a compound spectrum.

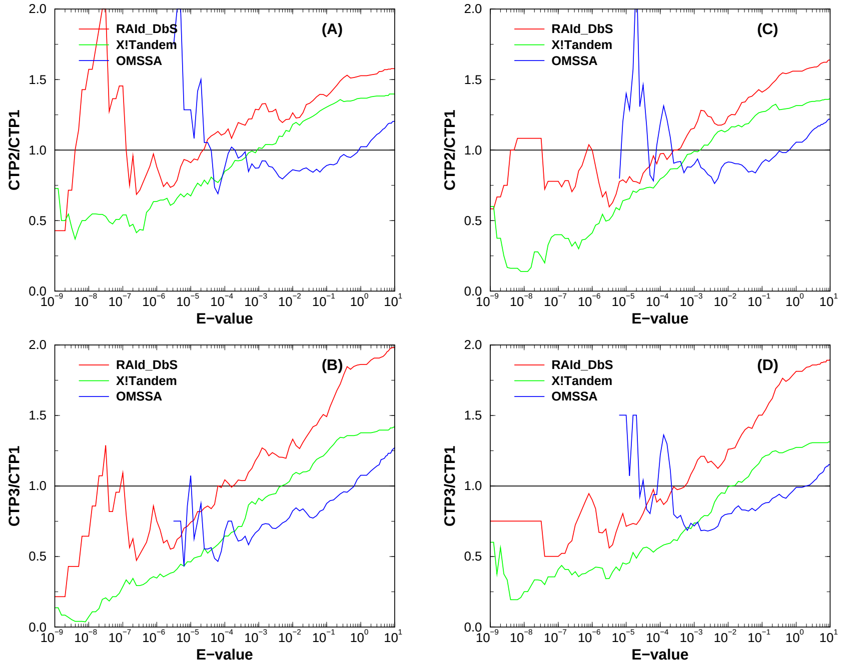

Figure 13: The cumulative identification ratio from analyzing compound spectra made by combining two (A) or three (B) TOF/TOF spectra in the co-identifiable set. The compound spectra are constructed using the SUM2 and SUM3 methods. Panels (C) and (D) show essentially the same content except that only unique-peptide spectra are combined to form compound spectra. The symbols CTP1, CTP2 and CTP3 correspond respectively to the cumulative number of true positives (TP) identified with E-value equal to or smaller than the specified cutoff when analyzing single-peptide spectra, compound spectra of two peptides, and compound spectra of three peptides.
